# Graphitic Carbon Nitride as a Platform for the Synthesis of Silver Nanoclusters

**DOI:** 10.1186/s11671-021-03621-z

**Published:** 2021-11-24

**Authors:** Halyna Starukh, Martin Koštejn, Vlastimil Matějka, Petr Praus

**Affiliations:** 1 Institute of Environmental Technology, CEET, VSB-Technical University of Ostrava, 17. listopadu 15, 70800 Ostrava-Poruba, Czech Republic; 2 Department of Chemistry, Faculty of Materials Science and Technology, VSB-Technical University of Ostrava, 17. listopadu 15, 708 00 Ostrava-Poruba, Czech Republic; 3 Chuiko Institute of Surface Chemistry of National Academy of Sciences of Ukraine, General Naumov Street 17, Kyiv, 03164 Ukraine; 4 Institute of Chemical Process Fundamentals, Czech Academy of Science, Rozvojová 1, 165 02 Prague, Czech Republic

**Keywords:** Graphitic carbon nitride, Silver nanoclusters, Synthesis, Photocatalysis, Ofloxacin

## Abstract

**Abstract:**

Graphitic carbon nitride (CN) synthetized by the thermal polycondensation of melamine at 550 °C for 4 h was further exfoliated by heating at 500 °C for 3 h. Silver cations were adsorbed on the exfoliated graphitic carbon nitride (CNE) and then reduced by sodium borohydride forming silver nanoclusters (NCs) with a size of less than 1 nm. The NCs were located on the CNE surface and did not change the CNE properties except for its pore size distribution and thereby specific surface area (SSA). The Ag NCs were able to collect the photoinduced electrons of CNE and thus reduce their recombination with the holes. It was also documented by the increase in the CNE photocatalytic activity in terms of the degradation of antibiotic Ofloxacin. This study demonstrates the ability of CNE to serve as a platform for a simple and fast synthesis of Ag NCs without any stabilizing compounds.

**Graphic Abstract:**

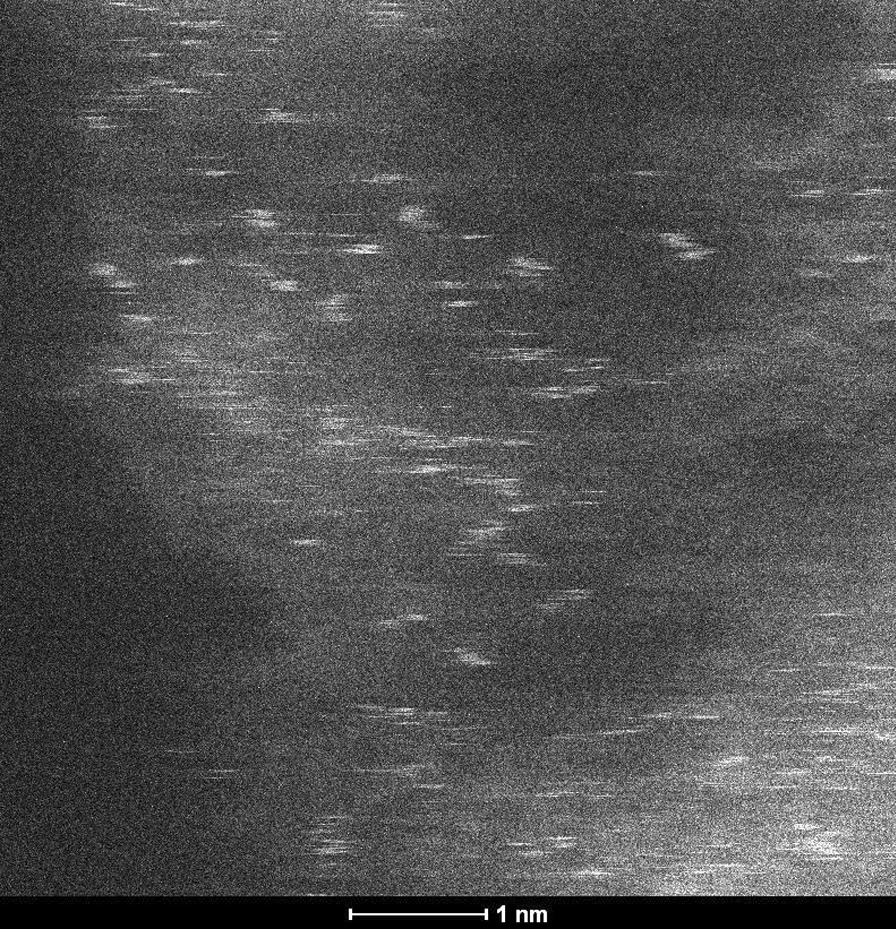

**Supplementary Information:**

The online version contains supplementary material available at 10.1186/s11671-021-03621-z.

## Introduction

Graphitic carbon nitride is a two-dimensional (2D) metal-free polymeric material of the theoretical composition CN with remarkable chemical and electronic properties. This metal-free semiconductor allows the absorption of visible light due to its band gap energy of 2.7 eV. Important properties, such as high thermal, physical, chemical and photochemical stability, allow for the application of CN for solar cell production, imaging and sensing of some compounds [11, 20, 44], 66, 81.

One drawback of CN photocatalysts is connected with the rapid recombination of photoinduced electrons and holes and low quantum efficiency (about 0.1% under irradiation with 420–460 nm light wavelength) [33]. Another drawback is its low SSA, which can, however, be increased by the thermal exfoliation of bulk CN [60] leading to the formation of nanosheets and their clusters. For example, the CN mesoporous material with an SSA of about 281 m^2^ g^−1^ can be obtained by the direct application of various platforms [61]. Additionally, a nitrogen-rich intervening space between tri-s-triazine units allows CN to have unique properties for the stabilization of metal single atoms, clusters or nanoparticles [25].

CN has been applied as a catalyst for a variety of reactions, such as the Biginelli [8] and Friedel–Crafts reactions of benzene [19], the Knoevenagel condensation [73] and the CO_2_ capture and conversion [62]. Over the last years, it has become an increasingly engrossing topic in the field of photocatalysis [21, 32, 69]. As an n-type semiconductor, CN demonstrates a serious possibility for realizing processes, such as hydrogen evolution [42, 53, 80, 82], CO_2_ reduction [23, 41] and organic pollutants degradation [34, 67].

Noble metal nanoparticles (NPs) demonstrate great potential for numerous applications in chemical, biological, electronic, medical and catalytic areas due to their exclusive properties [3, 4, 26], compared to their bulk analogues. For example, gold NPs are widely used for drug delivery, various biological applications and catalysis. The antimicrobial properties of silver NPs and their usage in photodynamic antimicrobial chemotherapy, promising capabilities in biosensors and in fuel cells [7, 31] are well-known as well.

The preparation of Ag NPs by the reduction of silver salts is a widely applicable procedure. The reducing agents very often include borohydride, sodium citrate, ascorbic acid, alcohols, hydrazine compounds and plant extracts. Many papers have been published on the synthesis and properties of Ag NPs so far, for example, [1, 28, 40, 43, 45, 54, 57, 65, 71, 77]. The NPs tend to agglomerate and have to be stabilized using various capping agents, such as various polymers and surfactants [30, 56]. Another approach is in using inert supports which promote the high dispersion of Ag NPs, preventing their agglomeration and stabilizing them. Some supports have been used for this purpose, such as SiO_2_, TiO_2_, ZrO_2_ [29], ZnO [13], montmorillonite [52], sepiolite [37], zeolite [22], metal–organic frameworks [9], nitrogen-doped carbon [46], macro-/mesoporous carbon [12] and graphitic carbon nitride [14] as well.

A special new nanoscience issue is the synthesis and stabilization of ultra-small metal nanoparticles, as well as metal nanoclusters. The synthesis of single atoms and atom clusters is of great importance for biosensing and detection, cancer and antibacterial therapy, catalysis, etc. [15, 35, 36, 58, 75].

The attempts to improve the photocatalytic activity of CN by Ag-doping and coupling with Ag compounds have been highly increased in recent years. The photoassisted reduction method was applied by Bu et al. [5] to prepare Ag-modified CN. The Ag NPs were synthesized by thermal treatment of Ag^+^ ions in a mixture with melamine and NH_4_OH [68] or by the deposition of Ag^+^ ions on CN followed by their reduction [18, 70], 72, 74.

Even if the idea of using CN as a platform for the deposition of nanoparticles is not quite new, because, for example, we also synthetized SnO_2_ nanoparticles in the presence of CN [49], the synthesis of Ag nanoparticles or Ag nanoclusters using CNE with high SSA and micro- and mesoporous structure has not yet been published. In this work, we applied CNE as a platform for the synthesis of Ag NCs without any stabilizing agent. The simple procedure was based on the adsorption of Ag^+^ ions on exfoliated CN followed by their reduction with NaBH_4_. The prepared nanomaterials were characterized by various methods including testing their photocatalytic activity by means of the degradation of Ofloxacin.

## Experimental

### Synthesis of Bulk and Exfoliated CN

Bulk CN was synthesized by the thermal polycondensation of melamine according to a previously reported method [48]. Typically, 10 g of analytical grade melamine powder was placed into a covered ceramic crucible at room temperature and heated in a muffle furnace in air. The temperature was gradually elevated with a heating rate of 3 °C min^−1^ and kept at 550 °C for 4 h. The crucible was removed from the furnace, cooled down to an ambient temperature and then ground in an agate mortar.

For the CN exfoliation, bulk CN placed on a ceramic plate was heated in a muffle furnace with a heating rate of 10 °C min^−1^ to 500 °C and kept at this temperature for 3 h. The exfoliated CN (labelled as CNE) was cooled down to an ambient temperature out of the oven.

### Adsorption of Ag^+^ on CNE

CNE was dispersed into an aqueous AgNO_3_ solution (100 mL) with a concentration of 0.5, 1.0, 2.5 and 5.0 mmol L^−1^, respectively, to obtain Ag^+^ deposited CNE materials with the various Ag loadings (0.1, 0.5, 1.0, 2.0 and 5.0 wt%). These suspensions were stirred over 2 h, after that the solids were separated by centrifugation. The concentration of Ag^+^ ions in filtrates was determined by a silver electrode (Theta 90, Czech Republic). The sample used for further analyses was labelled as Ag^+^CNE2.

### Reduction of Ag^+^ on CNE

The reduction of Ag^+^ ions adsorbed on CNE was performed with NaBH_4_ (molar ratio Ag/NaBH_4_ = 1:2). 15 mL of the NaBH_4_ solution was added to 35 mL of the aqueous Ag^+^ and CNE suspension under stirring. After 10 min of stirring, the solid part was centrifuged, thoroughly washed with distilled water and dried at 105 °C for 2 h. The samples were labelled as AgCNE1, AgCNE2, AgCNE3, AgCNE4.

### Determination of Ag

The silver content in the AgCNE nanomaterials was determined by an X-ray fluorescence spectrometer (XRF) SPECTRO Xepos (SPECTRO Analytical Instruments GmbH, Kleve, Germany).

### X-Ray Diffraction

X-ray diffraction (XRD) technique was used to study the phase composition and microstructural properties of the AgCNE nanomaterials. XRD patterns were obtained using a Rigaku SmartLab diffractometer Rigaku (Tokyo, Japan) with a detector D/teX Ultra 250. The source of X-ray irradiation was a Co tube (CoKα, *λ*_α1_ = 0.178892 nm, *λ*_α2_ = 0.179278 nm) operated at 40 kV and 40 mA. The XRD patterns were collected in the 2*θ* range of 5°–90° with the step size of 0.01° and the speed of 0.5° min^−1^. The calculation of crystallite size *L* was performed by the Debye–Scherrer formula:

1 $$B\left(2\theta \right)=\frac{K\lambda }{L{\mathrm{cos}}\theta }$$

where *θ* is Bragg’s angle, *λ* is the wavelength of X-rays, and *K* is the constant equal to 0.94 for cubic and 0.89 for spherical crystallites. In this work, *K* = 0.90.

### Fourier-Transform Infrared Spectrometry

Fourier-transform infrared spectrometry (FTIR) was performed using a Nicolet iS50 device (Thermo Scientific, Waltham, MA, USA). The FTIR spectra were collected in an attenuated total reflection (ATR) mode using a diamond ATR crystal. The spectra were collected in the wavenumber range of 400–4000 cm^−1^. The ATR correction followed by baseline subtracting was applied on each spectrum using the OMNIC software (Waltham, MA USA).

### Specific Surface Area Measurements

Specific surface area measurements were performed with a SORPTOMATIC 1990 device (Thermo Scientific, Waltham, MA, USA). SSA was determined by the analysis of N_2_ adsorption isotherms recorded at − 196 °C by means of the Brunauer–Emmett–Teller (BET) method. The Barrett–Joyner–Halenda (BJH) model was used for the calculation of pore size distributions.

### UV–Vis Diffuse Reflectance Spectrometry

UV–Vis diffuse reflectance spectra (DRS) were obtained with a spectrophotometer Shimadzu UV-2600 (IRS-2600Plus) in the range of 220–1000 nm. DRS was transformed to the Kubelka–Munk function F(R) as follows:

2 $$F{(R}_{\infty })=\frac{{\left(1-{R}_{\infty }\right)}^{2}}{{2R}_{\infty }}$$

where *R*_∞_ is the diffuse reflectance from a semi-infinite layer. The values of band gap energies (*E*_g_) were determined according to the well-known Tauc’s procedure [63].

### Transmission and Scanning Electron Microscopy

Transmission electron microscopy (TEM) was carried out using a JEOL 2100 microscope with a LaB6 electron gun. An accelerating voltage of 160 kV was applied. TEM micrographs were taken by a camera Tengra (EMSIS). The samples were prepared by their suspension in ethanol and sonication for 5 min. One drop of this suspension was placed on a copper grid with a holey carbon film and dried at room temperature.

High-resolution transmission electron microscopy (HRTEM) was carried out using a Titan G2 microscope (FEI) with an image corrector on the accelerating voltage of 80 kV. HRTEM micrographs were recorded with a BM UltraScan CCD camera (Gatan). Energy-dispersive spectrometry (EDS) was performed in a Scanning TEM (STEM) mode by a Super-X system with four silicon drift detectors (Bruker). STEM micrographs were taken with a high-angle annular dark-field (HAADF) detector 3000 (Fishione). The CNE materials were dispersed in methanol and sonicated for 5 min, and one drop was put on a copper grid with a holey carbon film. After drying in air at room temperature, the HRTEM analysis was performed.

Scanning electron microscopy (SEM) was performed using a microscope Hitachi SU6600 with the accelerating voltage of 5 kV, and the acquisition time was 300 s. Before the analysis, the samples were placed on a conductive carbon tape.

### X-Ray Photoelectron Spectroscopy

The superficial elemental analysis was performed by means of an X-ray photoelectron spectrometer (XPS) ESCA 3400 (Kratos Analytical Ltd., UK) with a base pressure in the analysis chamber of 5.0 × 10^7^ Pa. Electrons were excited with an Mg Kα radiation (*hν* = 1253.6 eV) generated at 12 kV and 10 mA. For all spectra, the Shirley background was subtracted. Peaks ascribed to the *sp*^2^ hybridized nitrogen (C=N–C) were set to 398.8 eV as a charge correction.

### Photoluminescence Spectrometry

Photoluminescence (PL) spectra were obtained with a spectrometer FLS920 (Edinburgh Instrument Ltd., UK). The spectrometer was equipped with a 450 W Xenon lamp (Xe900). The excitation wavelength was 325 nm.

### Photocatalytic Experiments

The photocatalytic activity of the AgCNE nanomaterials was investigated by means of the degradation of Ofloxacin in a concentration of 20 mg L^−1^. In the dark, 10 mg of each AgCNE material was added into 150 mL of the tested solution and stirred for 60 min to reach adsorption–desorption equilibrium. Then, the stirred suspension in a glass vessel of 250 mL with 52 mm in height and diameter of 90 mm was irradiated from the top with a LED source (420 nm, 7.1 mW cm^−2^); the temperature was kept at 20 °C.

The photocatalytic degradation of Ofloxacin was monitored by high-performance liquid chromatography (HPLC). For the HPLC separation, a Waters 2996 chromatograph with a photodiode array (PDA) detector (Waters Corporation Milford, MA, USA) and a Synergi 4 µm Polar-RP 80 Å (100 × 3 mm) column were used. The mobile phase consisted of acetonitrile and 0.04 mol L^−1^ chloroacetic acid (25:75, v/v), and pH = 3 was adjusted with NH_4_OH.

## Results and Discussion

The AgCNE materials were prepared by the adsorption of Ag^+^ ions on exfoliated graphitic carbon nitride and the reduction with NaBH_4_. As demonstrated in Table 1, the content of silver after the reduction was lower than the theoretical content of Ag^+^ likely due to the loss of metallic Ag which was not captured in CNE pores. Some physico-chemical properties of the prepared materials were characterized by the following methods.

**Table 1:** The content of Ag in AgCNE nanomaterials

Material	Ag theoretical (wt%)	Ag by XRF (wt%)
AgCNE1	0.5	0.29
AgCNE2	1.0	0.60
Ag^+^CNE2	1.0	0.89
AgCNE3	2.5	1.3
AgCNE4	5.0	2.4

### Structure and Texture Properties of AgCNE Nanomaterials

The structure of prepared AgCNE nanomaterials was studied by TEM, HRTEM, SEM, XRD, FTIR, XPS, UV–Vis DRS and PL spectrometry. Specific surface area and pore size distribution were analysed based on the physisorption of nitrogen. The photocatalytic activity was tested in terms of the degradation of Ofloxacin.

#### Analysis by Electron Transmission Microscopy

**Fig. 1:**
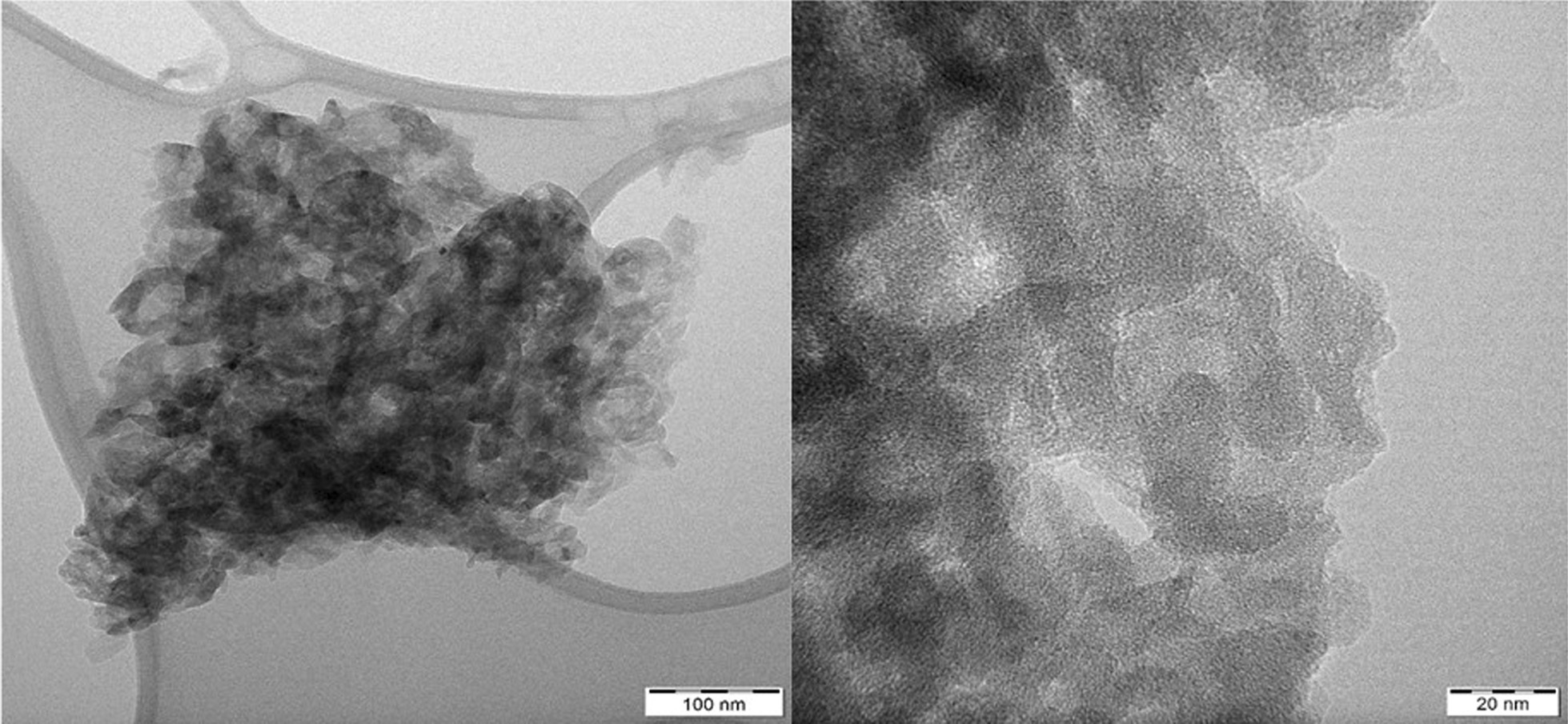
TEM micrographs of AgCNE4 with different resolutions

The morphology and microstructure of the prepared AgCNE nanomaterials were studied by electron microscopy. All these nanomaterials exhibited similar flake-like structures consisting of single flakes as well as their agglomerates. Figure 1 demonstrates the typical TEM micrograph of graphitic carbon nitride; no Ag nanosize objects are visible. In order to confirm their presence, an HRTEM analysis was performed as well.

**Fig. 2:**
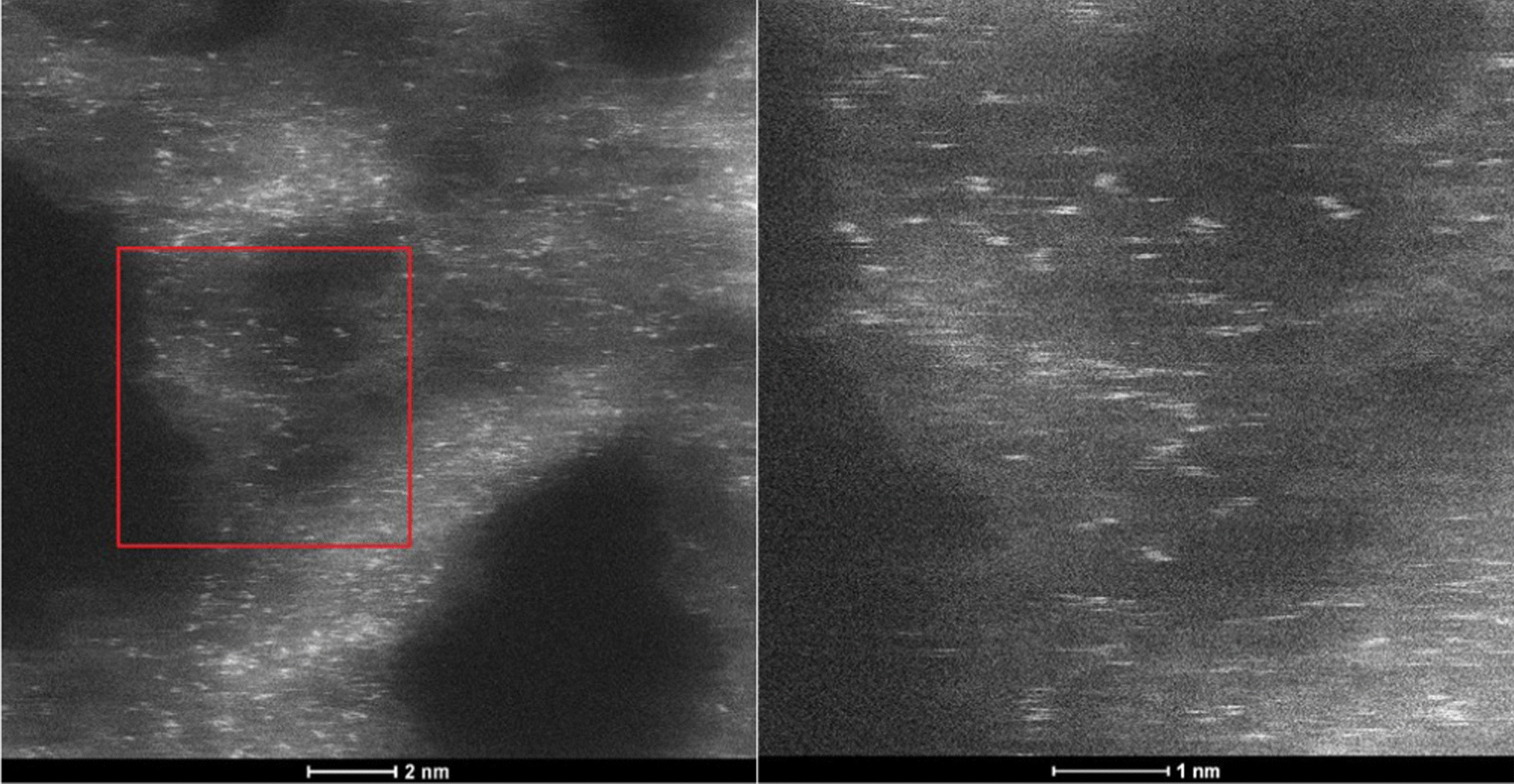
STEM micrographs of AgCNE4 (left) and its detail view (right)

The STEM micrographs of AgCNE4 in Fig. 2 show small Ag objects with a size equal to or less than 1 nm spread on the CNE surface. Such objects can be composed of several Ag atoms which are not arranged in a regular crystalline structure. That is the reason why no Ag diffractions were observed in the XRD patterns, see below (Fig. 4). These small objects can be considered as Ag nanoclusters, and therefore, the term “nanoclusters” was further used instead of “nanoparticles”.

**Fig. 3:**
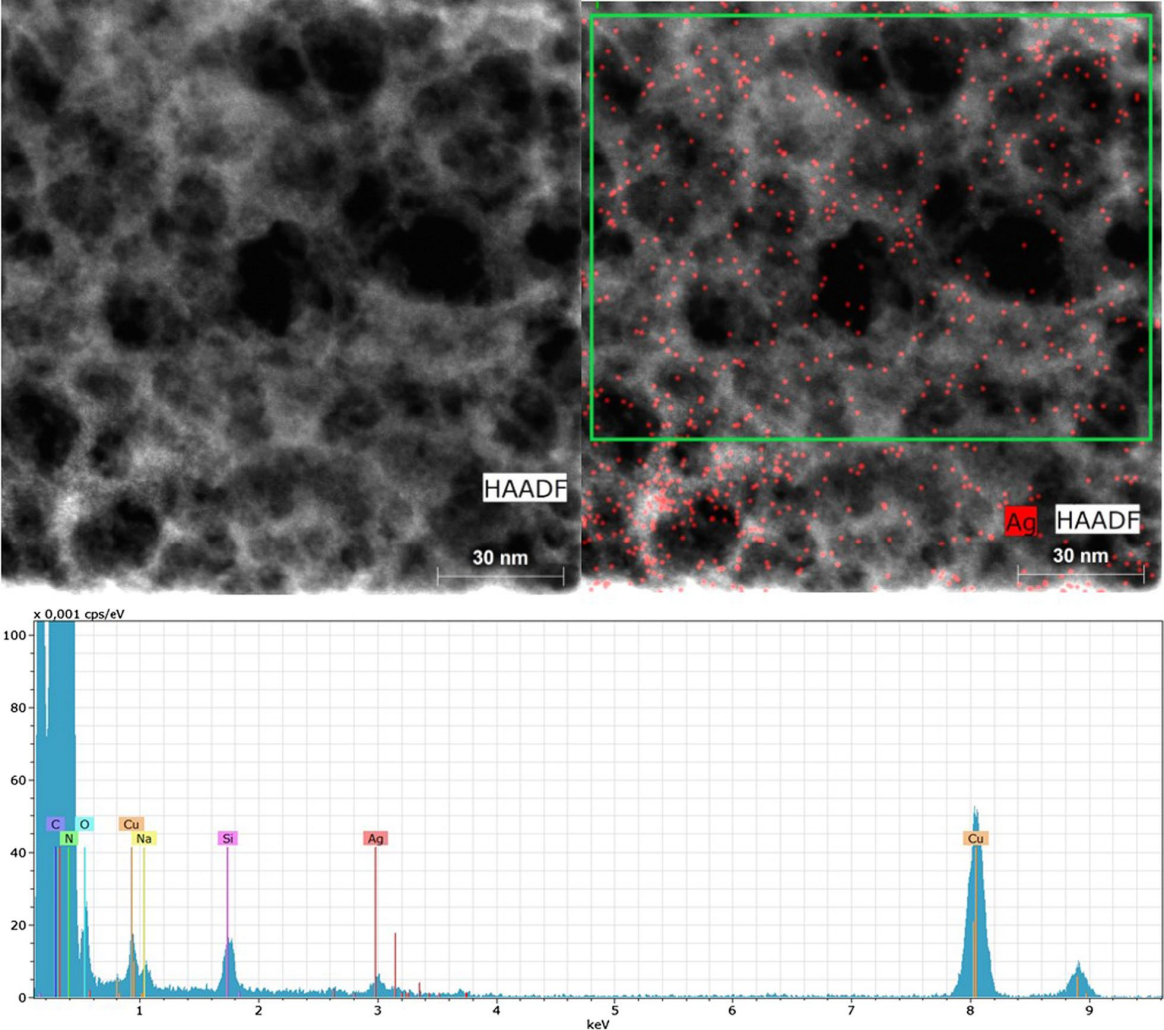
HAADF-STEM micrographs and EDS spectrum (down) of AgCNE4

The STEM analysis with the HAADF imaging (HAADF-STEM) is shown in Fig. 3. The Ag nanoclusters marked by red spots are well dispersed on the CNE surface. The EDS spectrum in Fig. 3 demonstrates the Kα lines of present elements. Some minor elements, such as Na and Si, were impurities coming from glassware. Cu was present due to using the copper grid, and the presence of O was due to the synthesis in air and the presence of adsorbed water. The EDS spectrum with the log scale of intensities (Additional file 1: Fig. S1), as well as the element compositions (Additional file 1: Table S1), is given in Supplementary materials. The content of Ag was estimated from the area marked by a green rectangle at 1.8 wt%, which is close to the value of 2.4 wt% found by XRF (Table 1). Moreover, the porous structure of AgCNE4 is visible as well.

#### XRD Analysis

**Fig. 4:**
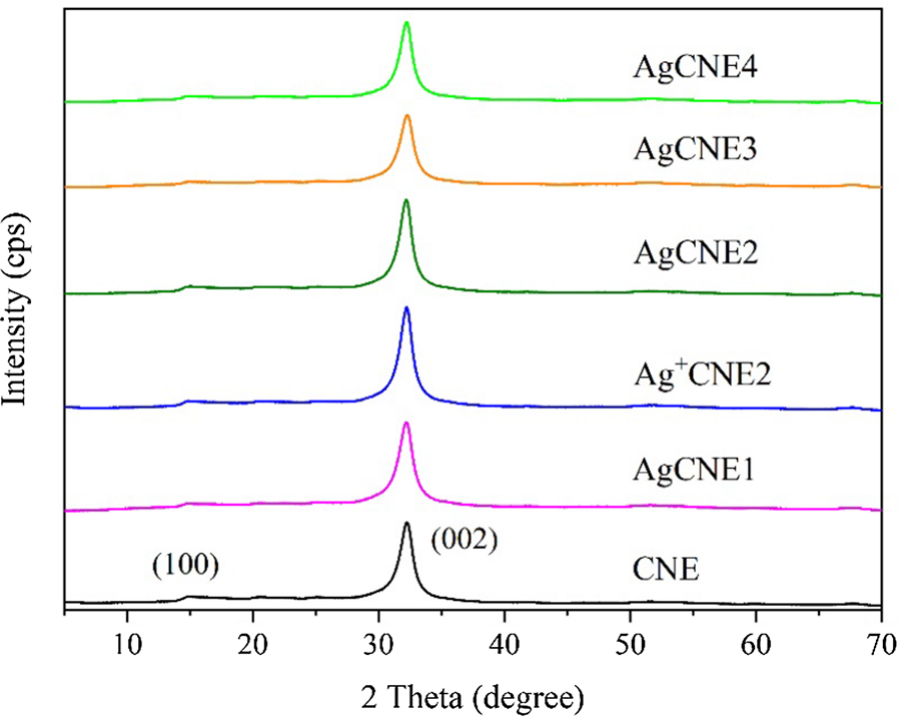
XRD patterns of AgCNE nanomaterials

The structure of synthesized AgCNE nanomaterials was studied by XRD analysis. The hexagonal phase of graphitic carbon nitride (JCPDS 87-1526) was confirmed for CNE by the presence of two diffraction peaks in the XRD patterns, see Fig. 4. The low intensive peak (100) at around 2*Θ* = 15° is ascribed to the in-plane ordering of nitrogen-linked heptazine units [64]. The more intensive peak (002) at 2*Θ* = 32.2° corresponds to the interlayer diffraction of the conjugated aromatic system of CNE. Due to the low intensive (100) peaks, the XRD patterns with log intensity coordinates were used for the detail peak description, see Additional file 1: Fig. S2 and Table 2.

**Table 2:** Two main diffraction peaks, crystallite sizes and interlayer distances of AgCNE nanomaterials

Nanomaterials	(100) 2Θ (°)	(002) 2Θ (°)	L(002) (nm)	d(002) (nm)
CNE	14.88	32.25	7.3	0.322
AgCNE1	14.88	32.17	6.9	0.323
Ag^+^CNE2	14.88	32.26	7.4	0.322
AgCNE2	15.03	32.21	7.4	0.322
AgCNE3	14.98	32.29	7.2	0.322
AgCNE4	14.88	32.24	7.4	0.322

These XRD patterns demonstrated the typical CN peaks (Fig. 4 and Additional file 1: Fig. S2), but no diffraction peaks of metallic silver were observed due to the low content of Ag and the small size of Ag NCs in contrast to our previous work concerning the bigger Ag NPs with the average size of 7 nm deposited on montmorillonite [52]. No influence of deposited Ag (Ag^+^) on the interlayer AgCNE distances was observed because the calculated crystallite sizes *L*(002) were very similar, 6.9–7.4 nm (Table 2). These findings indicate that no Ag NCs were intercalated between the CNE layers.

#### FTIR Analysis

**Fig. 5:**
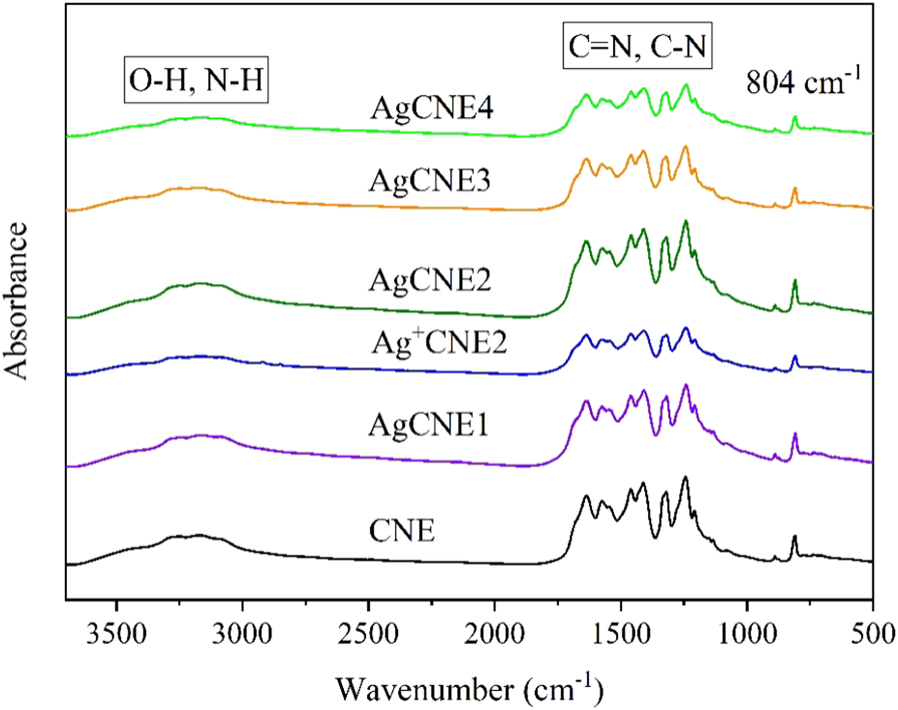
FTIR spectra of AgCNE nanomaterials

The surface chemical state of the AgCNE nanomaterials was studied with FTIR spectrometry, see Fig. 5. The spectral bands at 3100–3400 cm^−1^ correspond to the stretching vibration modes of N–H and O–H bonds which can be related to uncondensed amino groups and absorbed H_2_O molecules, respectively. The spectral bands at 1100–1680 cm^−1^ can be ascribed to the stretching vibrations of C=N and C–N bonds of heterocyclic rings. The medium band at 804 cm^−1^ corresponds to the breathing mode of tri-s-triazine ring moieties [10]. All these spectra are typical for graphitic carbon nitride, and their explanation is in good agreement with other reports on CN layered structures [47, 51].

All the FTIR spectra are very similar, which is well visible in their overly spectrum shown in Figure S3. No effects of deposited Ag NCs on the stretching and deformation vibrations mentioned above indicate that (1) the CNE structure was not influenced by Ag NCs and (2) the amount of deposited silver was small. Similar findings were observed by other authors [14, 39, 55, 59, 78]. Unlike [76], the interaction of Ag^+^ ions with CNE amino groups decreasing their N–H vibrational bands was not observed. The likely reason is the small amount of Ag^+^ as well.

#### XPS Analysis

The XPS analysis was conducted in order to study the valence states of Ag NCs. In addition, the XPS spectra of C, N and O, as well as their survey spectrum, were also recorded, but they showed common information of graphitic carbon nitride which can be found elsewhere [48]. These spectra are given in Supplementary materials, see Additional file 1: Figs. S4 to S7.

**Fig. 6:**
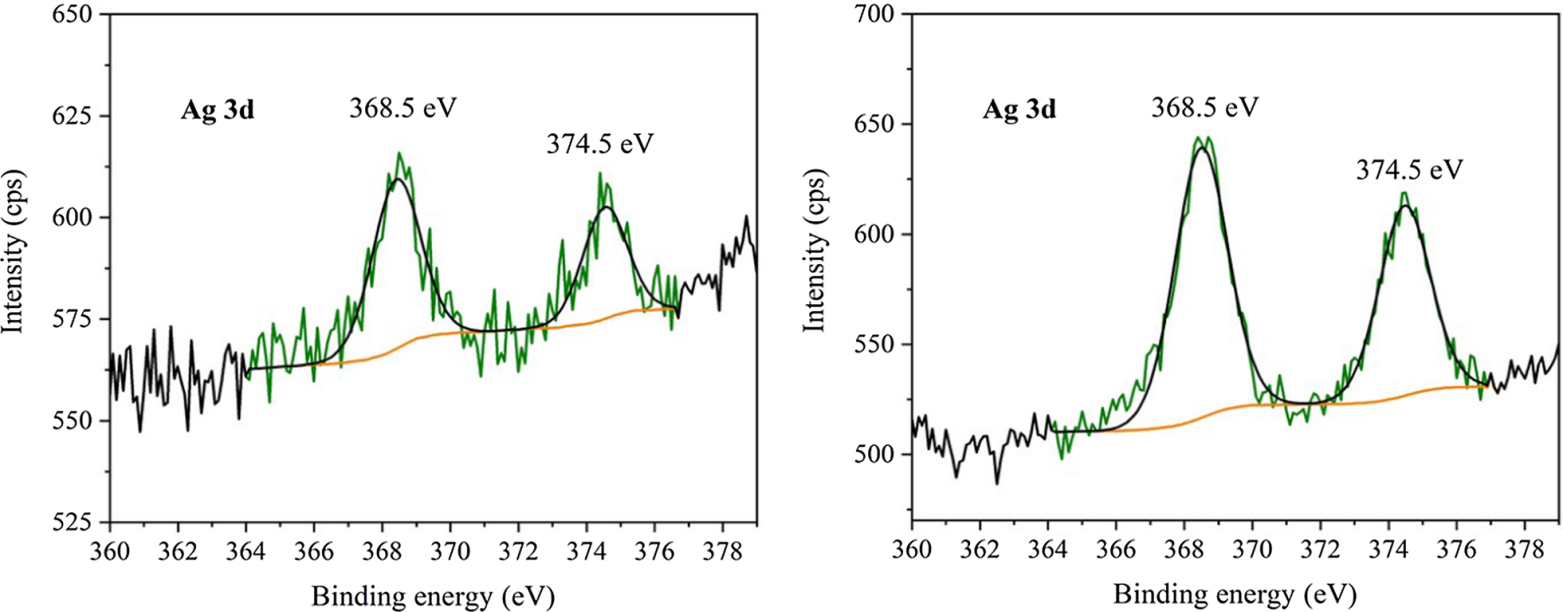
XPS spectra of Ag 3d of Ag^+^ adsorbed on CNE (left) and AgCNE4 (right)

Figure 6 shows the XPS spectrum of AgCNE4 with the highest content of silver. The peaks at 368.5 eV and 374.5 eV are typical for Ag 3*d*_5/2_ and Ag 3*d*_3/2_ [2, 27]. However, the same XPS spectrum was obtained for the CNE with adsorbed Ag^+^ ions without reduction, see Fig. 6.

**Fig. 7:**
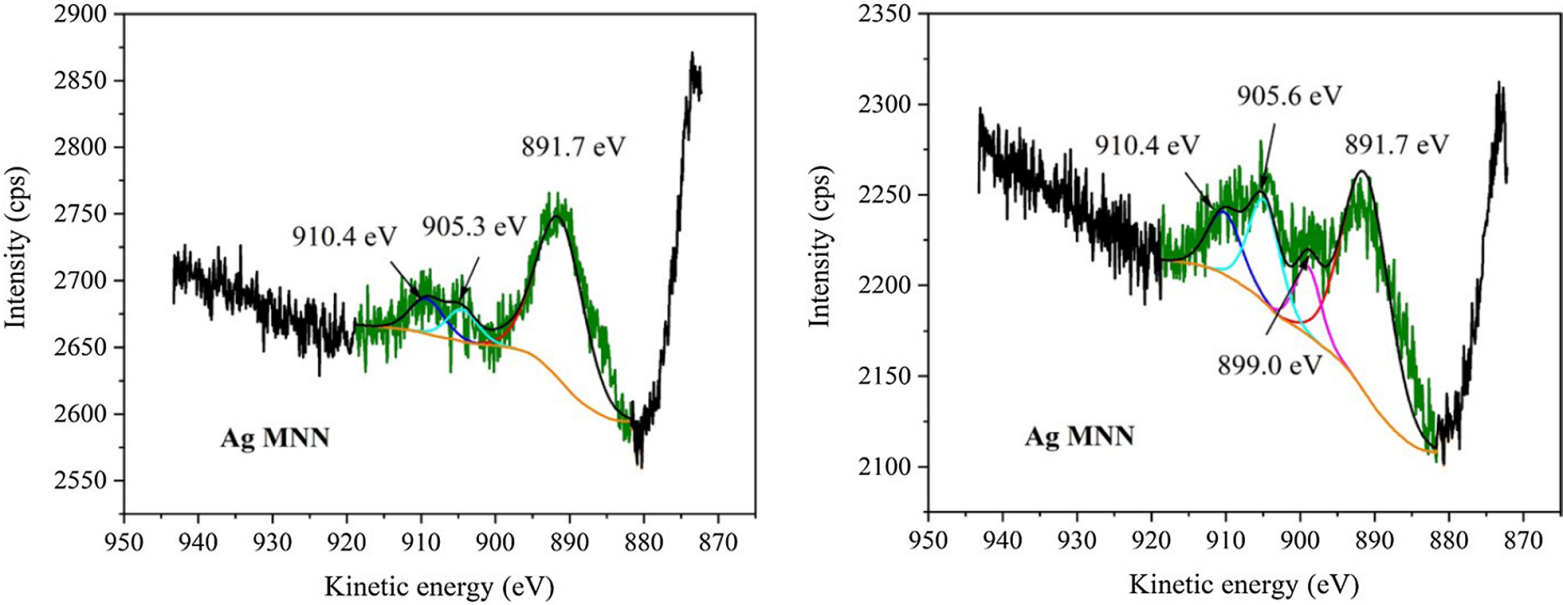
XPS spectra of Auger line Ag MNN of Ag^+^CNE2 (left) and AgCNE4 (right)

In order to identify the valence states of Ag in the metallic silver NCs, the Auger spectra of Ag MNN (transitions between the electron shells of atom) of AgCNE4 and Ag^+^ adsorbed on CNE were recorded, see Fig. 7. In all the spectra, the peak at 891.7 eV corresponding to N KLL was present. In these spectra, three peaks corresponding to Ag MNN were observed at 910.4 eV, 905.6 eV (905.3 eV) and 899.0 eV. The Ag MNN spectra are formed of double peaks which originate from M_4_N_45_N_45_ and M_5_N_45_N_45_ lines (Ferraria et al. [16]) exhibiting a specific peak to peak splitting and intensity ratio. In the case of Ag^+^CNE2, two peaks centred at 905.3 eV and 910.4 eV were ascribed to double peaks from oxidized Ag. It is not possible to differentiate between Ag^+^ and Ag^2+^ in oxides at these low concentrations.

For AgCNE4, the peak at 899.0 eV was ascribed to metallic Ag^0^ M_4_N_45_N_45_ line. The line M_5_N_45_N_45_ corresponding to Ag^0^ overlapped with the silver oxide line at 905.6 eV resulting in the higher intensity and shift of this peak in comparison with the spectra of Ag^+^CNE2. In the sample AgCNE4, it is visible that it contains metallic Ag; however, it also contains a portion of Ag^+^ representing likely oxidized Ag. As NaBH_4_ is a relatively strong reduction agent with a good solubility, it can be expected that all Ag^+^ ions were reduced. On the other hand, Ag is easy to oxidize, so it can be expected that the Ag NCs would be oxidized on their surface after the exposure to air; however, the cores of the nanoclusters remained metallic.

#### Texture Analysis

**Fig. 8:**
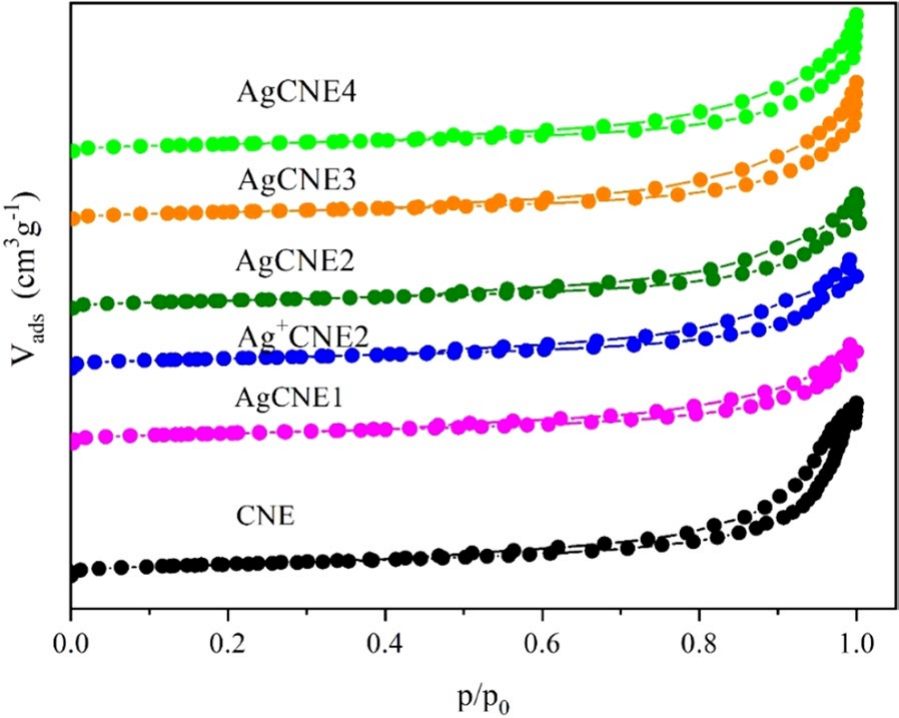
Nitrogen adsorption–desorption isotherms of AgCNE nanomaterials

**Fig. 9:**
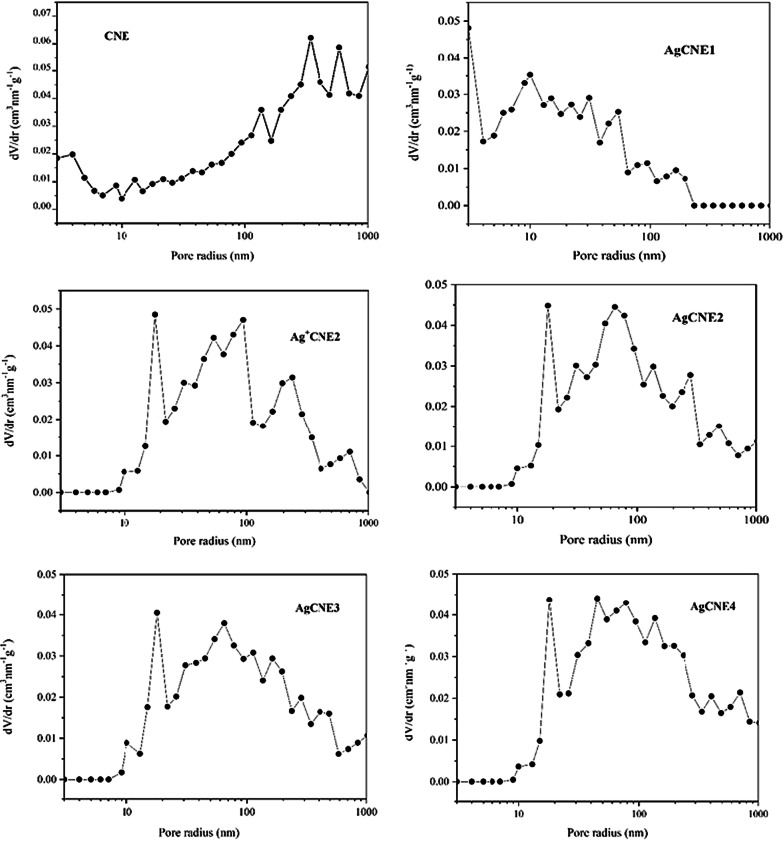
BJH pore size distribution curves of AgCNE nanomaterials

Nitrogen physisorption experiments were performed to study the texture properties of the AgCNE nanomaterials. The adsorption–desorption isotherms and pore size distribution curves are presented in Figs. 8 and 9, respectively. All the nanomaterials exhibited isotherms with hysteresis loops indicating the existence of slit-like mesopores formed likely under the overlapping and stacking of carbon nitride nanosheets.

The pore size distributions calculated from the nitrogen desorption isotherms by the BJH method show the broad range of pores due to the presence of mesopores and macropores (Fig. 9). It is remarkable that the presence of Ag nanoclusters, as well as that of Ag^+^, significantly changed the CNE pore size distribution by filling CNE pores, which resulted in the decrease in the specific surface area of CNE.

This effect is easily visible in the case of the CNE pores with radii up to 10 nm. It indicates that the CNE porous structure served as a platform for the synthesis of the Ag NCs. The pore size distributions of Ag^+^CNE2 and AgCNE2-AgCNE4 were similar. The specific surface areas are summarized in Table 3.

**Table 3:** The specific surface areas, pore sizes and band gap energies of AgCNE nanomaterials

Nanomaterials	Specific surface area (cm^2^g^−1^)	Band gap energy (eV)	k × 10^−3^ (min^−1^)
CNE	180	2.76	36.7 ± 6.1
Ag^+^CNE1	163	2.75	–
AgCNE1	157	2.77	31.7 ± 6.3
AgCNE2	151	2.77	43.4 ± 3.8
AgCNE3	168	2.77	38.7 ± 5.6
AgCNE4	149	2.78	33.1 ± 5.0

**Fig. 10:**
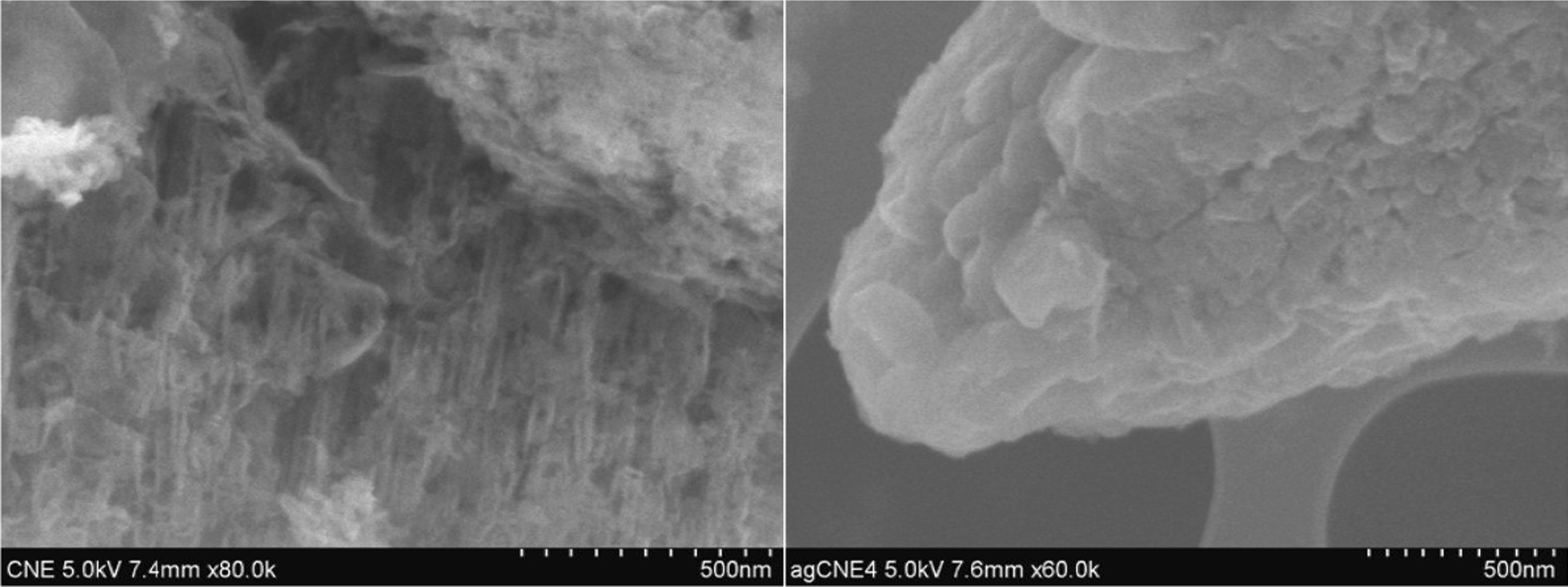
SEM micrographs of CNE (left) and AgCNE4 (right)

In order to demonstrate the pore distribution changes, the SEM micrographs of CNE and AgCNE were taken as shown in Fig. 10. The left micrograph displays the porous structure of CNE which was changed after the deposition of Ag NCs as shown in the right one.

### Optical Properties of AgCNE Nanomaterials

#### UV–Vis Absorption Study

**Fig. 11:**
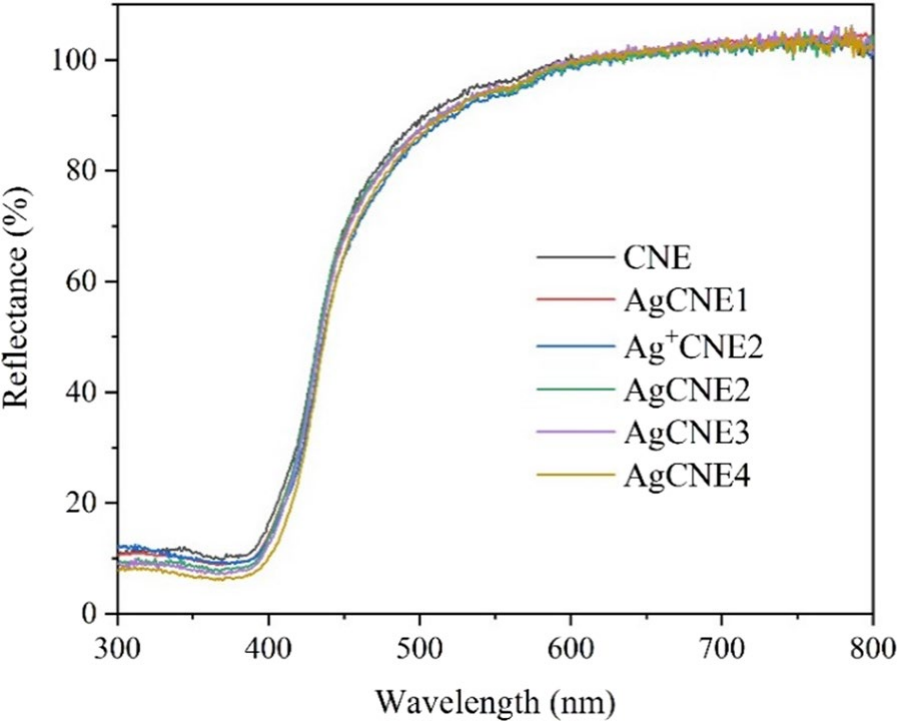
UV–Vis spectra of AgCNE nanomaterials

The optical properties of the AgCNE nanomaterials were examined by UV–Vis DRS. The presence of Ag NCs did not cause a significant change in the CNE optical absorption (Fig. 11) likely due to the low content of Ag. The band gap energies were determined according to common Tauc’s plots which were constructed from the UV–Vis DRS spectra. They are summarized in Table 3 and show no significant influence of the Ag NCs on the CNE band gap energy as well.

#### Photoluminescence Study

**Fig. 12:**
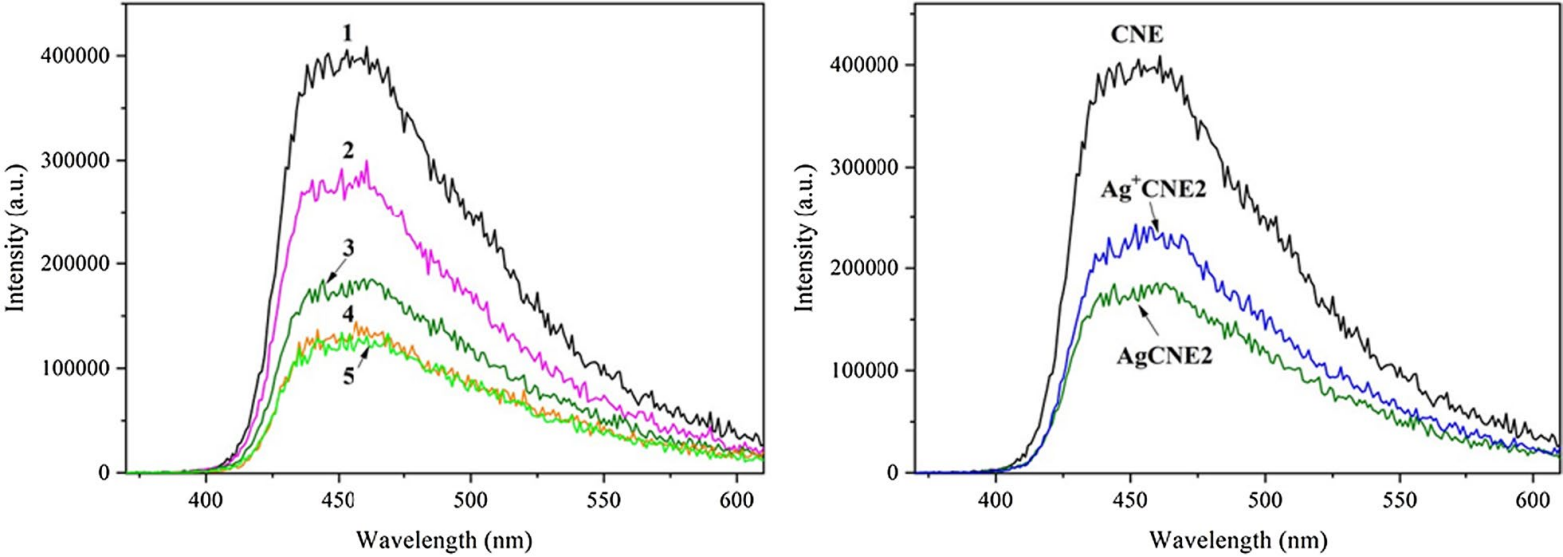
Photoluminescence spectra of AgCNE nanomaterials. Left—(1) CNE, (2) AgCNE1, (3) AgCNE2, (4) AgCNE3, (5) AgCNE4. Right—CNE, Ag^+^CNE2 and AgCNE2

The photoluminescence spectra were recorded to study the optical properties of the AgCNE nanomaterials as well, see Fig. 12. A strong and broad emission band from 400 to 600 nm with a maximum around 450 nm (2.76 eV) is related to multiple processes consisting of the band-to-band transition of photoinduced electrons and holes in CN [24], as well as the transition between lone pair and π* conduction band [79].

The emission peak intensity decreased with the increasing content of silver which can be explained by the transfer of electrons from CNE to the Ag NCs and, thus, the reduction of electrons–holes recombination [17, 38]. The PL spectra of AgCNE3 and AgCNE4 demonstrated a similar intensity which implies the silver loading probably did not play a role at these content levels. It should be mentioned that adsorbed Ag^+^ ions also caused the decrease in the PL intensity, likely due to the Ag^+^ ions reduction to metallic silver (Fig. 12).

### Photocatalytic Activity

**Fig. 13:**
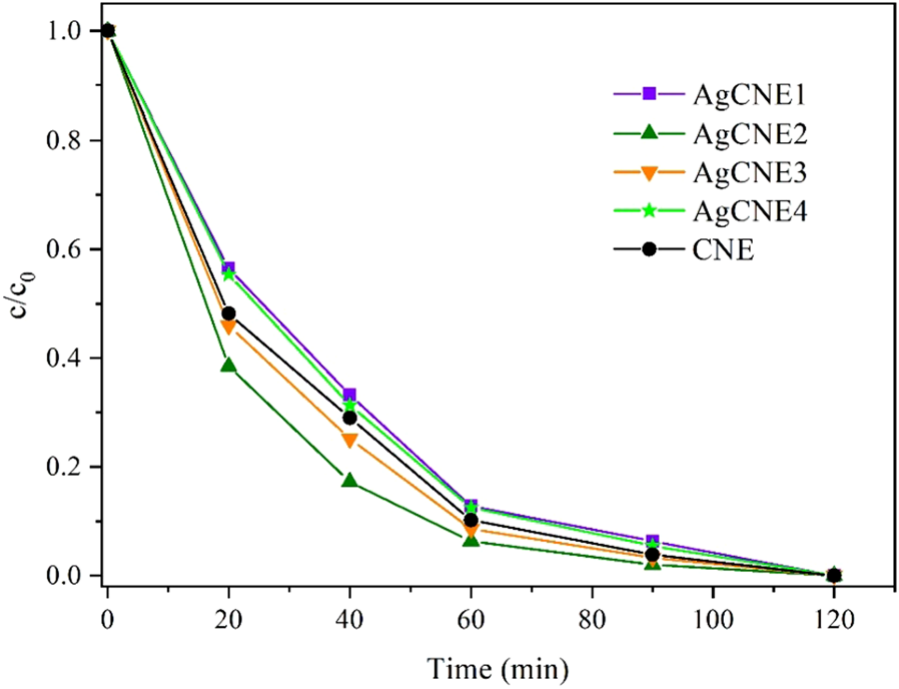
Photocatalytic degradation of Ofloxacin. LED source of 420 nm and 7.1 mW cm−2; the temperature was kept at 20 °C

The photocatalytic activity of the AgCNE nanomaterials was tested using the antibiotic Ofloxacin as shown in Fig. 13. No photolysis of Ofloxacin was observed. The reaction rate constants (*k*) were calculated according to the first-order reaction model as

3 $${\mathrm{ln}}\frac{c}{{c}_{0}}=-k\tau$$

where *τ* is the time, and c_0_ and c are the concentrations of Ofloxacin at *τ* = 0 and *τ* = *τ*, respectively. The rate constants are summarized in Table 3. They decrease in the order AgCNE2 > AgCNE3 ≥ CNE > AgCNE4 ≥ AgCNE1. It is obvious that in the cases of AgCNE2 and AgCNE3, the ability of CNE for the photocatalytic degradation of Ofloxacin can be further enhanced by the loading of silver nanoclusters. On the other hand, in the cases AgCN1 and AgCN4, the added silver NCs decreased the CNE activity.

As mentioned above, the Ag nanoclusters worked as collectors of photoinduced electrons and allowed them to be separated from holes, which led to the decrease in PL intensity [6]. The decreasing PL should be associated with an increasing photocatalytic activity, but it does not explain the above-mentioned order of the decreasing rate constants from AgCNE2 to AgCNE1. Comparing the photocatalytic activities with CNE, the smallest and the highest content of Ag reduced the photoactivity likely due to the shielding of the CNE surface covered with Ag NCs [50]. In the case of AgCNE2 and AgCNE3, the Ag NCs enhanced the photocatalytic activity of CNE due to the reduced recombination of photoinduced electrons and holes.

## Conclusion

Bulk graphitic carbon nitride was synthetized by the thermal polycondensation of melamine at 550 °C for 4 h and then was exfoliated by its heating at 500 °C for 3 h. Silver cations were adsorbed on the exfoliated graphitic carbon nitride (SSA of 180 m^2 ^g^−1^) and then reduced by sodium borohydride. In this way, the silver nanoclusters with a size of less than 1 nm were formed as observed by HRTEM. They were located on the CNE surface and substantially changed the pore size distribution of CNE. However, the Ag NCs were not intercalated between the CNE layers. The Ag NCs were likely partially oxidized due to their small size and reactive surface as indicated by XPS.

The formation of bonds between the Ag NCs and CNE was not observed in the FTIR and XPS spectra as well. The band gap energy of CNE (2.75–2.78 eV) was not changed by the silver presence as observed by UV–Vis DRS. The photoluminescence spectra indicated that the silver nanoclusters were able to collect photoinduced electrons of CNE and thus to reduce their recombination with holes. This effect was confirmed by testing the photocatalytic activity of the AgCNE nanomaterials. The Ag NCs enhanced the photocatalytic degradation of Ofloxacin in comparison with bare CNE.

The exfoliated graphitic carbon nitride was found to be the suitable platform for the synthesis of Ag nanoclusters with no need of further stabilization by means of chemical compounds. The synthesis of other metal nanoclusters in the presence of CNE for various applications of the resulting composite nanomaterials will be investigated in future research.

## Supplementary Information

**Additional file 1**. Graphitic Carbon Nitride as a Platform for the Synthesis of Silver Nanoclusters.

## Data Availability

Not applicable.

## Abbreviations

CN: Graphitic carbon nitride
CNE: Exfoliated graphitic carbon nitride
NCs: Nanoclusters
SSA: Specific surface area
2D: Two-dimensional
NPs: Nanoparticles
XRF: X-ray fluorescence spectrometry
XRD: X-ray diffraction
FTIR: Fourier-transform infrared spectrometry
ATR: Attenuated total reflection
BET: Brunauer–Emmett–Teller
BJH: Barrett–Joyner–Halenda
DRS: Diffuse reflectance spectrometry
TEM: Transmission electron microscopy
HRTEM: High-resolution transmission electron microscopy
EDS: Energy-dispersive spectrometry
STEM: Scanning transmission electron microscopy
HAADF: High-angle annular dark-field
XPS: X-ray photoelectron spectrometry
JPSD: Joint committee on powder diffraction standards
PL: Photoluminescence
LED: Light-emitting diode
HPLC: High-performance liquid chromatography
PDA: Photodiode array
AgCNE: Ag deposited on CNE
Ag^+^CNE: Ag^+^ deposited on CNE
