# Supplementary material for: Graphitic Carbon Nitride as a Platform for the Synthesis of Silver Nanoclusters
Source: Nanoscale Res Lett. 2021 Nov 24;16:166. doi: 10.1186/s11671-021-03621-z (PMC8613329; doi:10.1186/s11671-021-03621-z)
Supplement: Supplementary file 1 — Additional file 1. Graphitic Carbon Nitride as a Platform for the Synthesis of Silver Nanoclusters. [file 11671_2021_3621_MOESM1_ESM.docx]

**Supplementary materials**

**Graphitic Carbon Nitride as a Platform for the Synthesis of Silver Nanoclusters**

Halina Starukh^1,2,3^, Martin Koštejn^4^, Vlastimil Matějka^2^ and Petr Praus^1,2^

^1^Institute of Environmental Technology, CEET, VŠB-Technical University of Ostrava, 17. listopadu 15, 70800 Ostrava-Poruba, Czech Republic

^2^Department of Chemistry, Faculty of Materials Science and Technology, VŠB-Technical University of Ostrava, 17. listopadu 15,708 00 Ostrava-Poruba, Czech Republic

^3^Chuiko Institute of Surface Chemistry of National Academy of Sciences of Ukraine, General Naumov Street 17, Kyiv 03164, Ukraine

^4^Institute of Chemical Process Fundamentals, Czech Academy of Science, Rozvojová 1, 165 02 Prague, Czech Republic

Table S1 Elemental composition of AgCNE4

| Element | Content (wt. %) | Content (at. %) | Error (wt. %) |
| --- | --- | --- | --- |
| Silver | 1.84 | 0.22 | 0.92 |
| Nitrogen | 37.67 | 35.21 | 3.59 |
| Carbon | 57.53 | 62.71 | 5.47 |
| Oxygen | 1.27 | 1.04 | 0.26 |
| Silicon | 1.33 | 0.62 | 0.21 |
| Sodium | 0.37 | 0.21 | 0.15 |


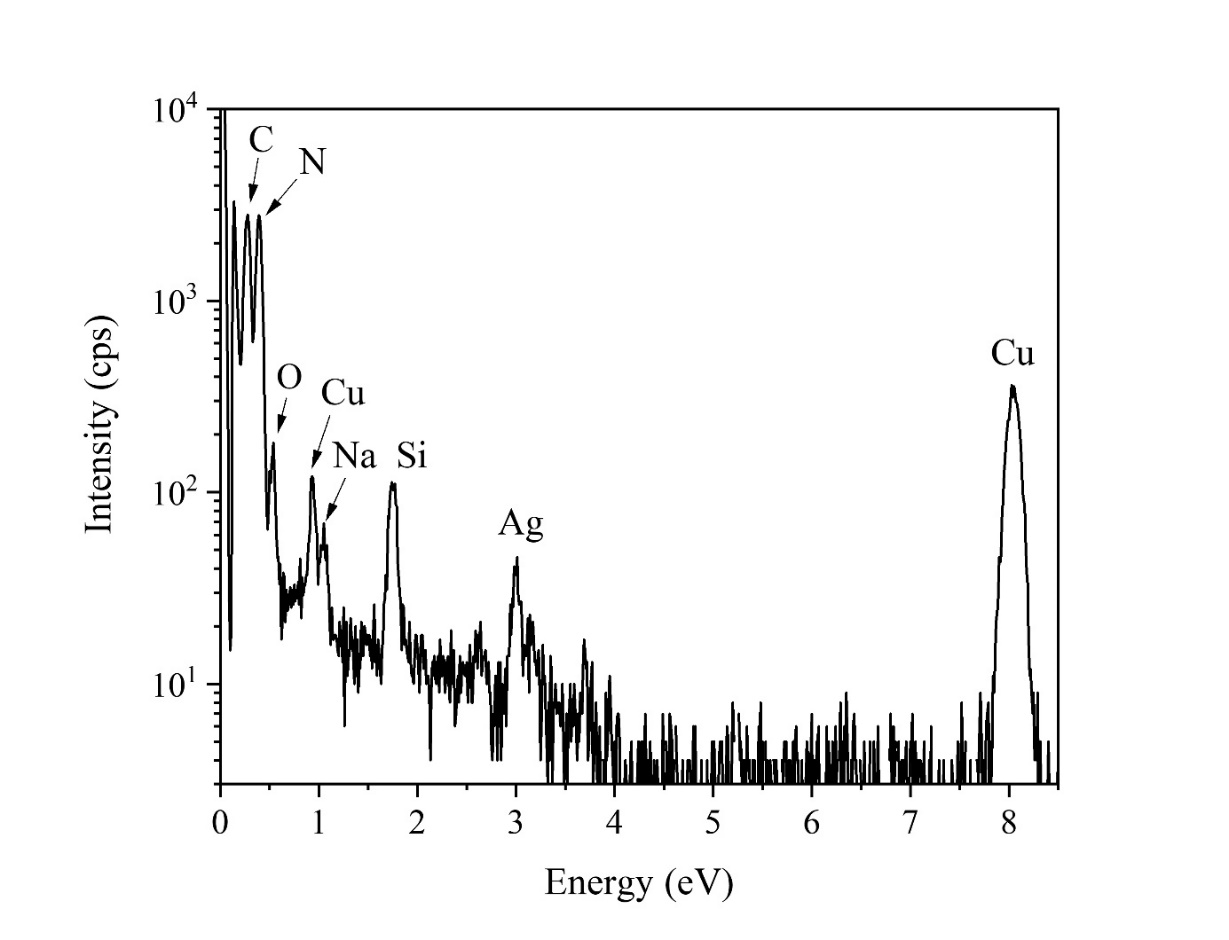


Fig. S1 EDS spectrum of AgCNE4.


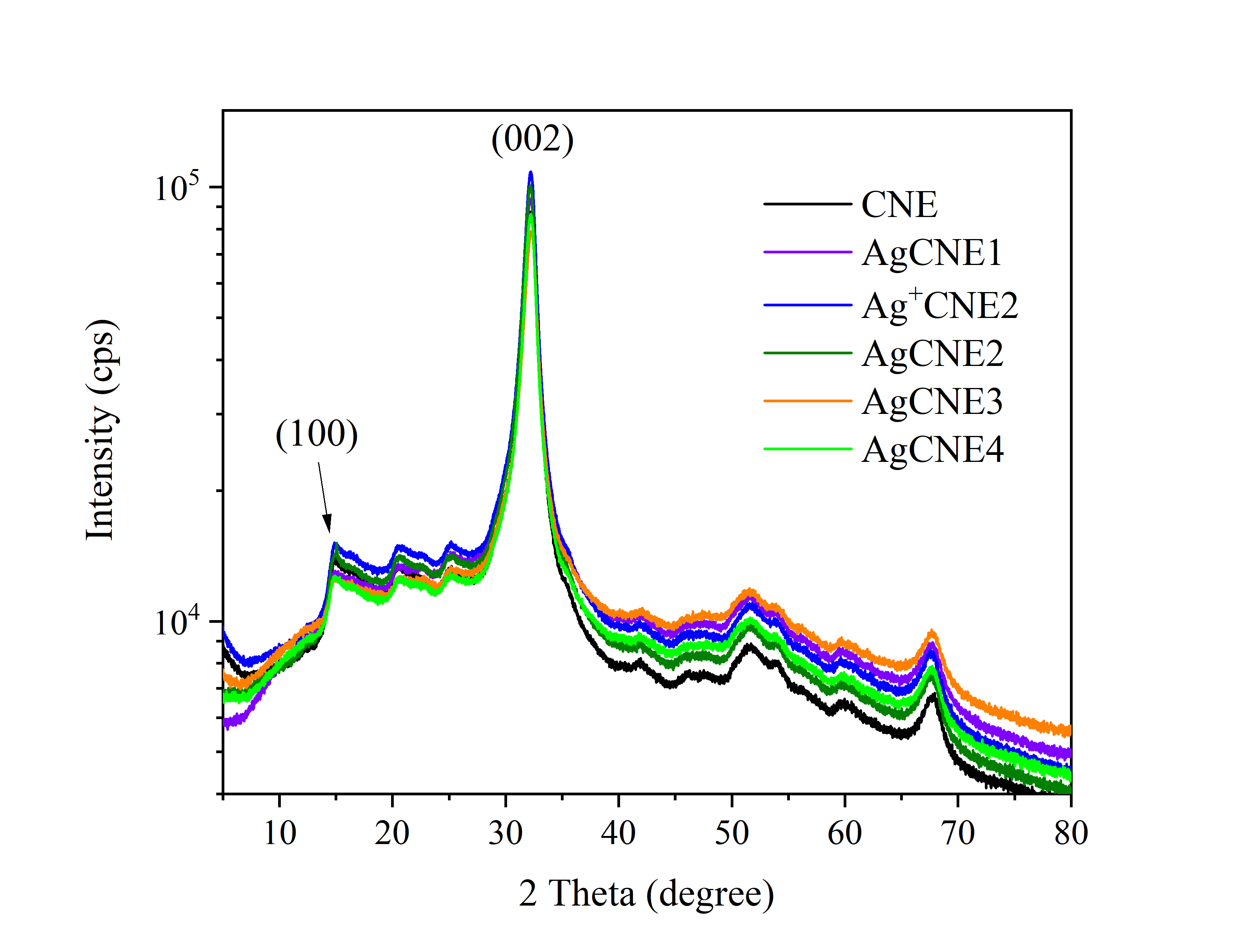


Fig. S2 XRD patterns of AgCNE nanomaterials.


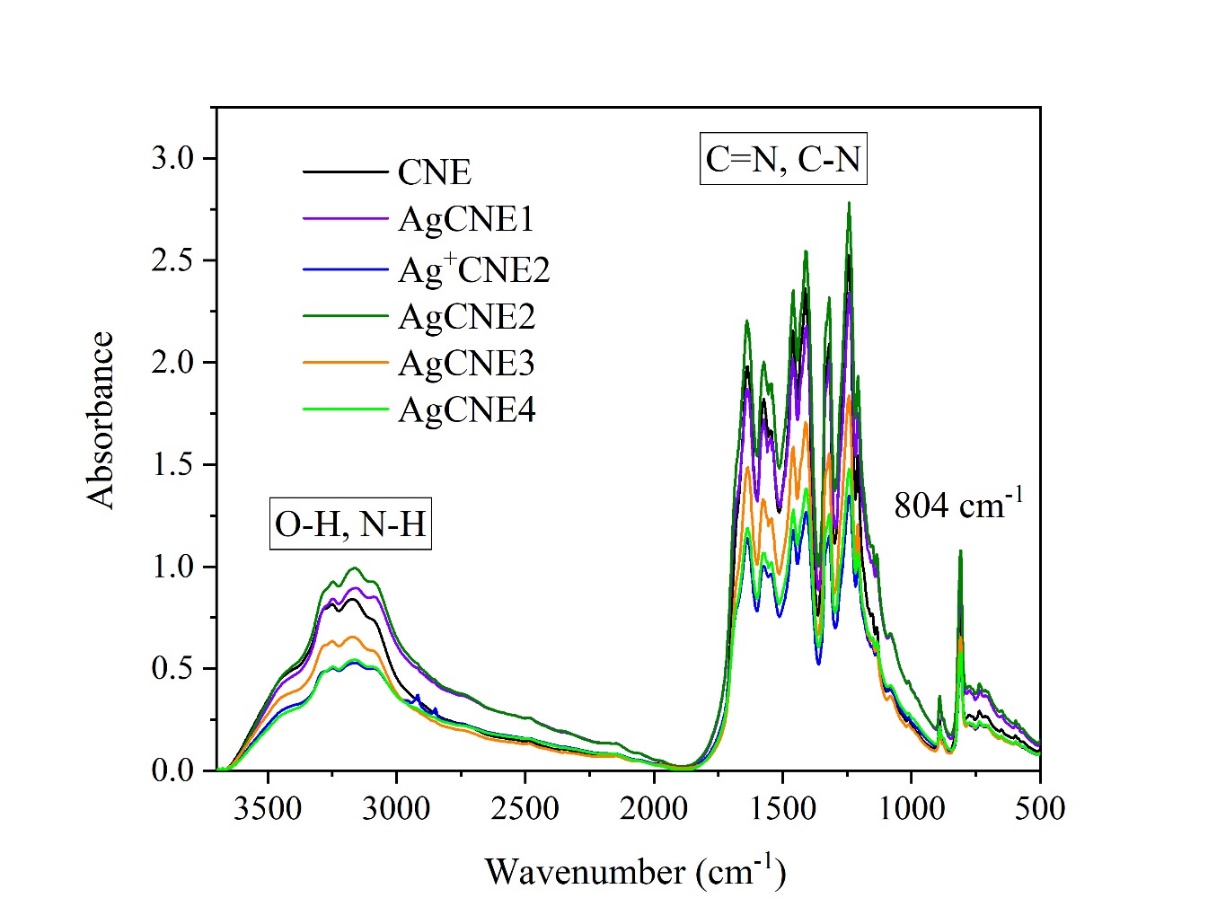


Fig. S3 FTIR spectra of AgCNE nanomaterials.


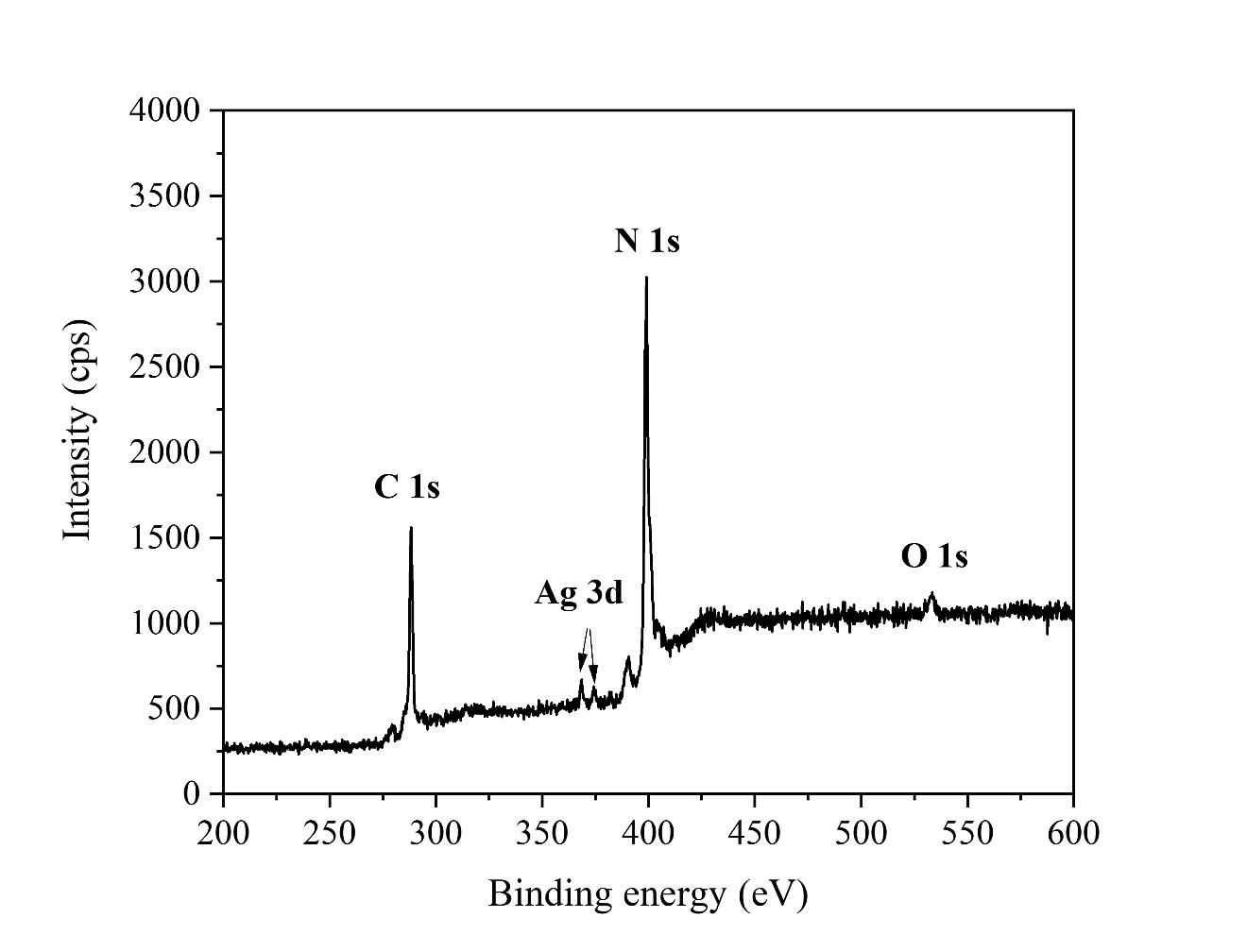


Fig. S4. XPS survey spectrum of AgCNE4.


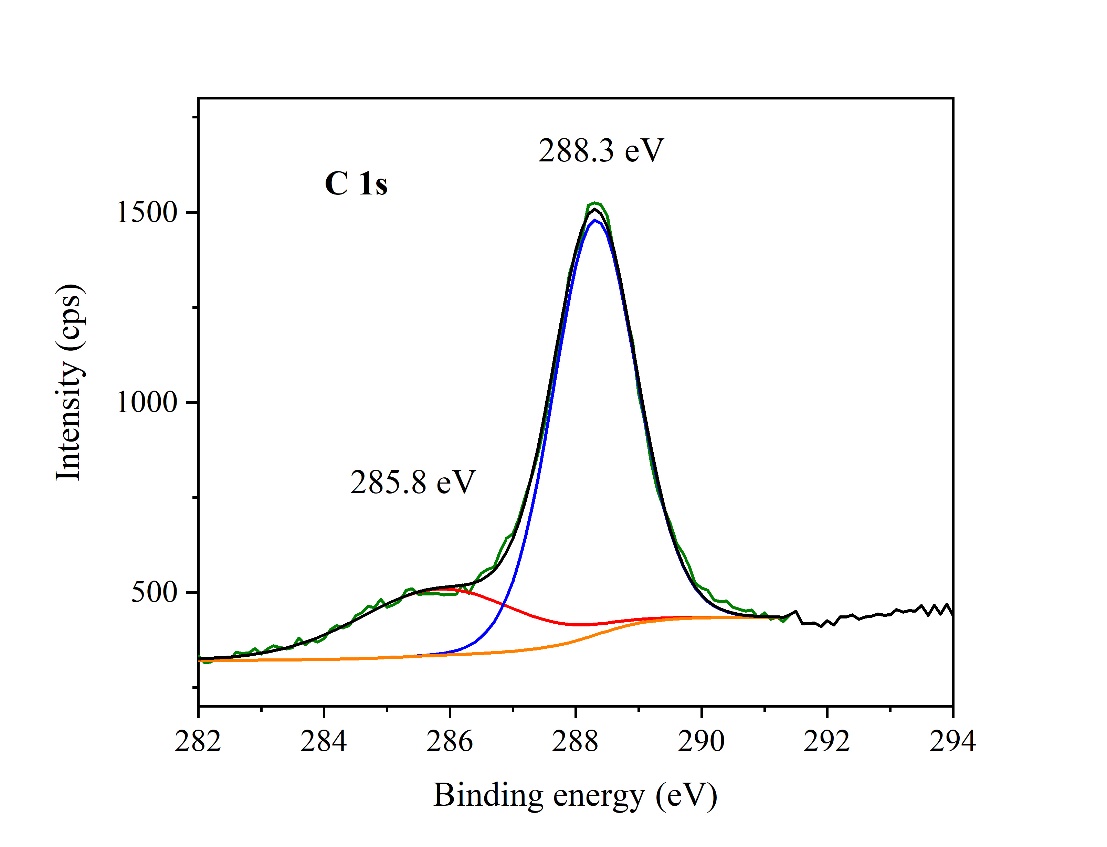


Fig. S5. XPS spectrum of C 1s of AgCNE4.


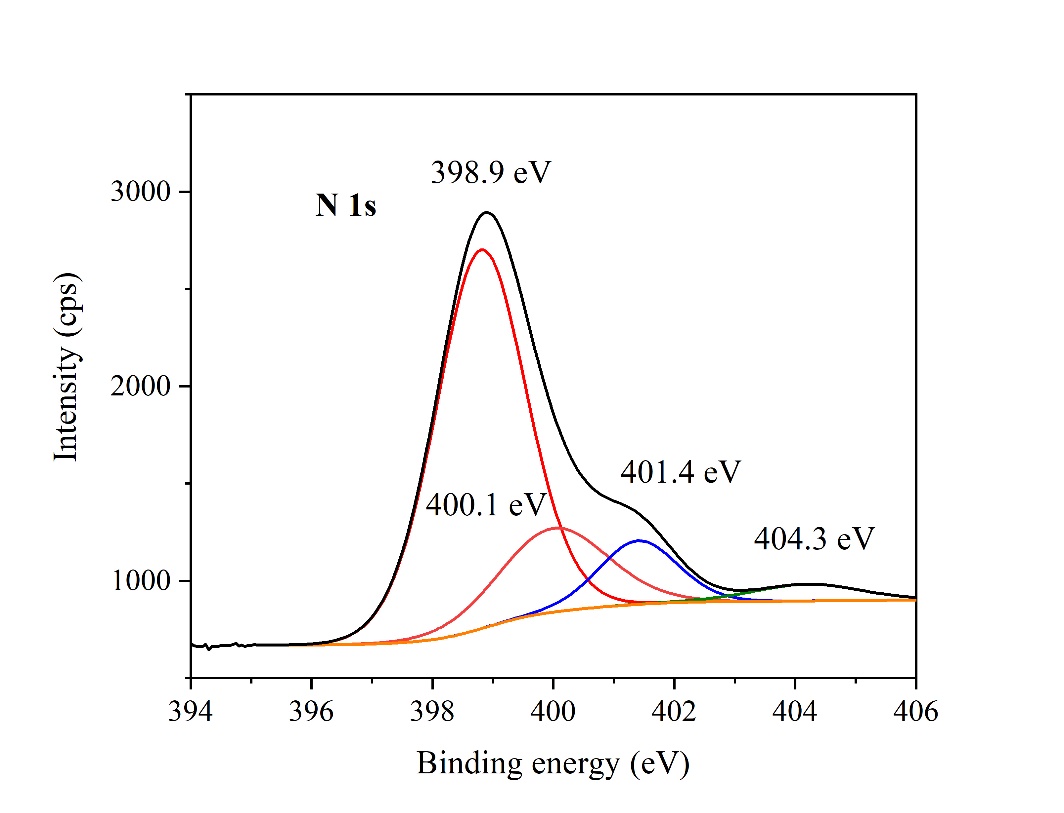


Fig. S6. XPS spectrum of N 1s of AgCNE4.


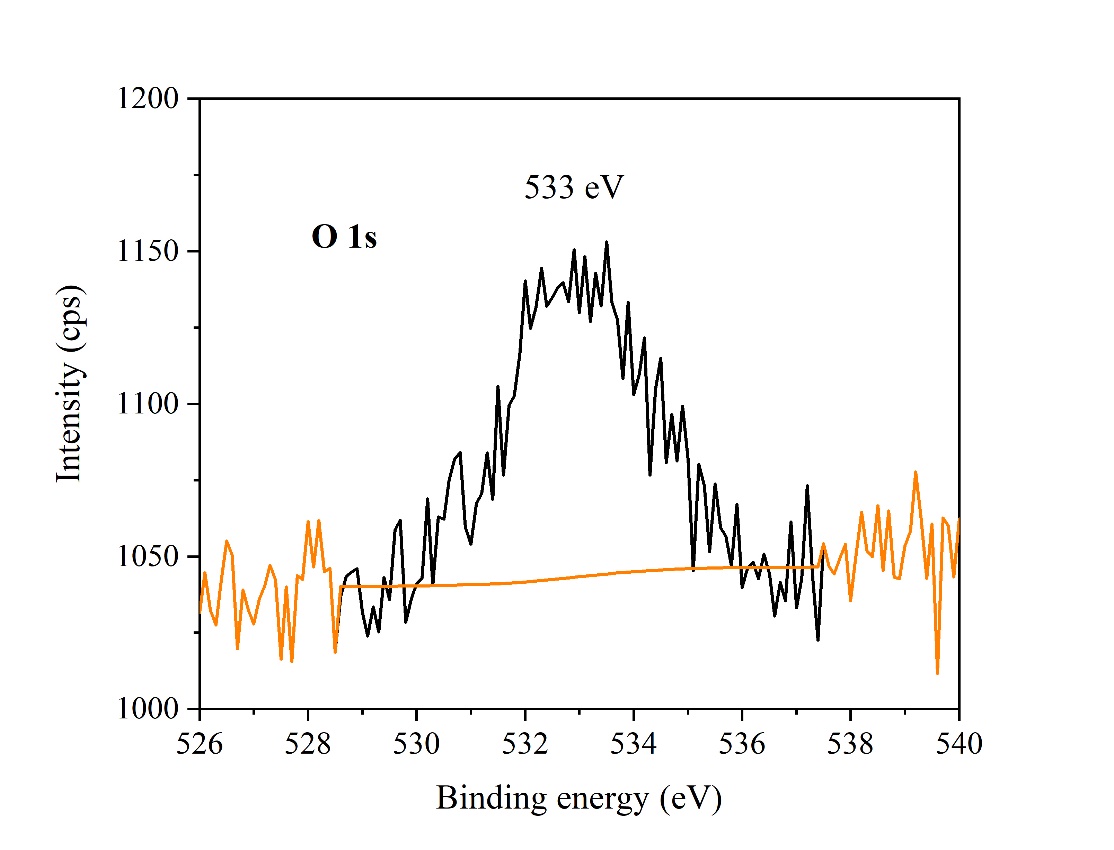


Fig. S7. XPS spectrum of O 1s of AgCNE4.
